# The modulatory action of C-Vx substance on the immune system in COVID-19

**DOI:** 10.1080/22221751.2022.2125347

**Published:** 2022-11-04

**Authors:** Ilhan Tahrali, Nilgun Akdeniz, Vuslat Yilmaz, Umut C. Kucuksezer, Fatma B. Oktelik, Ozkan Ozdemir, Esin Cetin-Aktas, Yelda Ogutmen, Arzu Ergen, Neslihan Abaci, Erdem Tuzun, Oral Oncul, Gunnur Deniz

**Affiliations:** a Department of Immunology, Istanbul University, Aziz Sancar Institute of Experimental Medicine, Istanbul, Türkiye; b Department of Neuroscience, Istanbul University, Aziz Sancar Institute of Experimental Medicine, Istanbul, Türkiye; c Faculty of Medicine, Department of Medical Genetics, Acibadem Mehmet Ali Aydinlar University, Istanbul, Türkiye; d Istanbul Faculty of Medicine, Department of Infectious Diseases and Clinical Microbiology, Istanbul University, Istanbul, Türkiye; e Department of Molecular Medicine, Istanbul University, Aziz Sancar Institute of Experimental Medicine, Istanbul, Türkiye; f Department of Genetics, Istanbul University, Aziz Sancar Institute of Experimental Medicine, Istanbul, Türkiye

**Keywords:** C-Vx, COVID-19, cytokines, cytotoxicity, immune system, inflammation, lymphocytes, proliferation

## Abstract

The modulatory effect of C-Vx, a novel therapeutic agent, on the immune system of COVID-19 patients was investigated. The functions of T and NK cells of COVID-19 patients with different disease severity were evaluated by flow cytometry in response to C-Vx stimulation. The levels of pro- and anti-inflammatory cytokines were detected by multiplex assay in supernatants after cell culture with C-Vx. Bradykinin, IRF3, and IFN-α levels were also measured by ELISA in the presence or absence of C-Vx stimulation. As a result, increased CD107a expression was observed on NK cells in response to C-Vx addition. The proliferation of T cell subsets was increased by C-Vx, decreasing by disease severity. IL-4 and IL-10 levels were elevated while IFN-γ and IL-17 levels were reduced in T cells following C-Vx stimulation. However, the levels of pro-inflammatory IL-1β, IL-6, IL-8, IFN-γ and GM-CSF were significantly increased upon C-Vx stimulation. IFN-α levels tended to increase after incubation with C-Vx. These findings support an immunomodulatory action of C-Vx on the immune system of patients with a mild and moderate phase of COVID-19.

## Introduction

Severe Acute Respiratory Syndrome-Coronavirus-2 (SARS-CoV)-2, the cause of Coronavirus Disease (COVID)-19, is an extremely contagious virus and a large part of the population is vulnerable to the infection with this virus. It has affected over 530 million people and caused the death of 6 million people worldwide to date.

SARS-CoV-2 can lead both to asymptomatic carriers as well as to patients expressing various pathognomonics from mild, moderate, and severe conditions – even death [1,2]. Hematological (lymphocyte and neutrophil count), inflammatory (C-reactive protein (CRP), procalcitonin (PCT)), and biochemical (D-dimer and ferritin) biomarkers which are critical for diagnosis and treatment, have been identified in association with COVID-19 [3].

After being infected with SARS-CoV-2, the immune system essentially launches a “combat” with the pathogen. Specifically, CD8^+^ T and natural killer (NK) cells as primary cytotoxic lymphocytes, have a critical role in the management of infections by mediating cellular immunity and cytotoxicity [4,5]. Recent studies have shown a decreased number of T and NK cells in severe cases, and the exhaustion and reduced functional diversity of T cells could contribute to the progression of COVID-19 [6–8]. On the other hand, excessive immune responses might lead to serious complications of the disease including inflammation-induced lung injury, respiratory failure, and death [9,10].

So far, there is no current specific and effective treatment for COVID-19. Along with the vaccines which have been developed to date, various therapeutic methods are also being investigated against COVID-19. In this context, it is considered important to redesign or improve existing natural and/or pharmaceutical treatments to deal with the virus threat.

The main ingredient of C-Vx was originally formulated for cancer treatment by Pharma-USA in conjunction with Miracle Labs Pharmaceutical Industry (unpublished data), and with the emergence of the COVID-19 pandemic, the scientific team has made modifications in the formula. Currently, C-Vx is patented as an active immunostimulant product (Patent Number: 17/497, 295). C-Vx is a repurposed vaccine combination containing anti-viral adjuvants and vitamins against SARS-CoV-2. The measles virus vector, which is used therapeutically against cancer as it replicates preferentially in and destroys cancer cells, and activates anti-tumour immune responses [11], was used also in C-Vx. According to the latest research, measles, mumps, and rubella vaccines might prevent or reduce the severity of COVID-19 [11,12]. Thus, bits of mumps and rubella viruses were added to the formula to improve the impact of C-Vx against SARS-CoV-2. The unique and novel formulation of C-Vx is expected to provide effective concomitant therapy against active COVID-19 infection.

The prophylactic use of products is thought to be a useful therapeutic approach for the prevention or treatment of the disease [13]. Thus, treatment opportunities for COVID-19 are much needed. This study aimed to explore the immunological aspects of the novel C-Vx substance and its possible contribution to the prevention and treatment of COVID-19. In order to see the action of the C-Vx as an immunomodulatory substance in COVID-19 patients, we aimed to better characterize and understand the status and functionality of immune cells by *in vitro* assays.

## Material and methods

### Study design and participants

COVID-19 patients [(*n* = 31); male (*n* = 15) and female (*n* = 16)] admitted to the pandemic clinic of Istanbul University, Istanbul Faculty of Medicine were enrolled in this study. All patients enrolled in this study were directed to laboratory immediately after clinically diagnosed and PCR-positivity was confirmed, they had COVID-19 for the first time and neither of the patients were vaccinated against SARS-CoV-2. This study was completed before the mutant strains spread to Turkey. Patients were grouped according to the clinical course (Table 1) as follows: Mild patients (*n* = 10), who are not hospitalized but have mild symptoms such as fever, dry cough; Moderate patients (*n* = 11), who have moderate/severe pneumonia or mild pneumonia with elevated clinical parameters; Severe patients (*n* = 10), who are hospitalized and have pneumonia with hypoxemia, severe clinical picture such as coagulation defects and multiple organ failure. The biochemical parameters of patient groups are given in Table 2. Age- and gender-matched healthy volunteers [(*n* = 10); male (*n* = 6) and female (*n* = 4)] without any known disease were also included in the study. Donors were not vaccinated against SARS-CoV-2 at the time of sampling. An approval of Istanbul University, Istanbul Faculty of Medicine, Clinical Research Ethics Committee was obtained (approval number 2020/1576) in compliance with the Helsinki declaration, while all donors signed informed consent forms prior to blood sampling.

**Table 1.** Demographic characteristics of COVID-19 patients and treatments they received.

	Mild	Moderate	Severe
N	10	11	10
Gender	5 males	5 males	5 males
	5 females	6 females	5 females
Age Median (Min-Max)	45 (21-79)	54.8 (33-78)	63.2 (50-73)
Treatment received for COVID-19	Hydroxychloroquine, Azithromycin	Hydroxychloroquine, Azithromycin, Favipravir	Hydroxychloroquine, Azithromycin, Favipravir, Tociluzumab
The clinical symptoms of COVID-19 patients	Who do not require hospitalization with Fever, sore throat, dry cough, malaise, and body aches or nausea, vomiting, abdominal pain, loose stools	Who have moderate/severe pneumonia or mild pneumonia without hypoxemia with high ferritin levels high – C reactive protein (hsCRP) levels, lymphopenia, hypotension dyspnea	Who are hospitalized pneumonia with hypoxemia (SpO_2_< 92%) with coagulation defects, encephalopathy, heart failure, and acute kidney injury

**Table 2.** Biochemical parameters of patients.

	Mild Median (min-max)	Moderate Median (min-max)	Severe Median (min-max)	Reverences Values
White Blood Cells (10³/µL)	8.03 (3.9-13.2)	6.6 (3.4-11)	10.4 (5-16.6)	4-10
Lymphocytes (10³/µL)	1.55 (0.6-3.1)	1.26 (0.5-2.4)	1.35 (0.2-5.9)	1.2-3.6
Neutrophils (10³/µL)	5.88 (2.1-11.8)	4.96 (1.7-10.1)	7.93 (0.6-15.4)	1.3-7
Platelets (10³/µL)	276.3 (177-528)	238.1 (124-364)	261.9 (123-476)	160-390
CRP (mg/dL)	40.1 (0-164)	58.9 (3-150)	153.7 (13-528)	0-5
D-Dimer (µg/L)	968.9 (190-3700)	922.7 (220-1640)	4370 (380-18450)	0.0-550
Ferritin (ng/mL)	192.9 (22-842)	572.3 (24-1404)	6068.6 (232-46852)	13-400
Procalcitonin (ng/mL)	0.08 (0.01-0.19)	0.1 (0.01-0.35)	10.97 (0.07-48.89)	0-0.5

### Sample preparation

Peripheral blood mononuclear cells (PBMCs) were isolated from heparinized peripheral blood samples by density gradient centrifugation using Ficoll-Paque (Histopaque-1077; Biochrom, Cambridge, UK). Following centrifuge at 2000 rpm for 20 min, collected PBMCs were washed with phosphate-buffered saline (PBS) and suspended in RPMI-1640 medium (Sigma Chem. Co., Germany). PBMCs were freshly used in culture assays and plasma samples separated from heparinized blood samples were frozen at −20°C until use in further assays.

In order to determine the non-toxic concentration of C-Vx, PBMCs (1 × 10^6^/mL) of healthy donors were cultured at different concentrations of C-Vx (1/50, 1/100, and 1/250) for 24, 48, and 72 h at 37°C incubator. After the cell culture, the apoptotic index was measured by using Annexin V-Apoptosis Detection Kit I (Biolegend, San Jose, CA, USA) and analyzed on flow cytometry. An optimal dose of C-Vx was determined as 1/250 and used in all culture assays (accepted in JCMR).

### Intracellular cytokine measurement

In order to determine the intracellular cytokine levels of T lymphocytes, PBMCs (1 × 10^6^ cells/mL) were pre-cultured with the presence or absence of C-Vx for 72 h and further stimulated with Cell Stimulation Cocktail [PMA (40.5 μM), Ionomycin (669.3 μM), and Brefeldin A (2.5 mg/mL)] (Biolegend, San Diego, USA) for 4 h at 37°C incubator. After the culture, cell surface staining was performed using anti-human-CD3-BV785, -CD4-PE, and -CD8-FITC monoclonal antibodies (mAbs) (all from Biolegend, San Diego, USA), prior to the detection of intracellular cytokine levels. Intracellular staining was performed using Fixation/Permeabilization Kit (BD Cytofix/Cytoperm, California, USA) according to the manufacturer’s protocol. Simply, cells were fixed and then permeabilized together with the addition of anti-IFN-γ-PE/Cy7, -TNF-α-APC/Cy7, -IL-4-APC, -IL-10-BV421, -IL-17-Alexa Fluor 700 mAbs. After washing, samples were measured by flow cytometry.

### The determination of NK cell cytotoxic activity

Pre-cultured PBMCs (5 × 10^5^) with or without C-Vx for 72 h, were stained with anti-human-CD107a-APC mAb (Biolegend, San Jose, CA, USA) and cultured alone or together with K562 cells (5 × 10^4^) at a 10:1 effector/target (E:T) ratio for 5 h at 37°C incubator. After the culture, PBMCs were labelled with anti-human-CD56-BV711, -CD16-BV570, -CD3-BV785, and -CD8-FITC (Biolegend, San Jose, CA, USA) mAbs. In order to determine the levels of intracellular cytotoxic granules; perforin and granzyme B, samples were fixed and permeabilized according to the manufacturer’s directions (Cytofix&Cytoperm Kit, BD Biosciences, San Jose, CA, USA), and stained with anti-human-Perforin-PerCp/Cy5.5 and -Granzyme B-Alexa Fluor 700 (Biolegend, San Jose, CA, USA) mAbs. Stained cells were measured and analyzed by flow cytometry.

### Lymphocyte proliferation assay by CFSE

To evaluate the proliferative responses of lymphocytes to C-Vx, carboxyfluorescein succinimidyl ester (CFSE) dilution method was used which is based on labelling cells with CFSE and evaluating the fluorescence halved by each cell division. PBMCs (up to 2 × 10^7^), freshly isolated and suspended in RPMI-1640 medium (Gibco, Paisley, UK), were stained with 1 μl of 5 mM CFSE solution (Thermo Fisher Scientific, USA) and incubated for 6 min at 4°C. Following washing with PBS, PBMCs were cultured for 120 h at 37°C with or without C-Vx (Miracle Labs Pharmaceutical Industry-Turkey) together with the absence or presence of 5 µl/mL PHA (Thermo Fisher, USA). Following cell culture, supernatants were collected and stored at −20°C for further analysis. PBMCs were harvested from the respective wells (US: unstimulated, PHA: phytohemagglutinin-stimulated, C-Vx, and PHA + C-Vx) into separate tubes for cell surface staining with anti-human-CD3-BV785, -CD4-PE-Cy7, -CD8-APC/Cy7, -CD16-BV570, and -CD56-BV711 (all from Biolegend, USA) mAbs. After the incubation, stained cells were washed with PBS and analyzed by flow cytometry.

### Detection of bradykinin, IRF3, and IFN-α levels by ELISA

Frozen plasma samples as well as supernatants of cultured PBMCs were thawed. The levels of bradykinin (sensitivity: 0.05 ng/mL, range: 0.2–6 ng/mL), IRF3 (sensitivity: 0.1 ng/mL, range: 0.312–20 ng/mL), and IFN-α (sensitivity: 0.5 pg/mL, range: 1.6–100 pg/mL) (AFG Bioscience, USA) were measured by ELISA according to the manufacturer’s instructions. Optical densities were measured at 450 nm and concentrations were calculated by reference to the standard curves.

### Cytokine measurement by multiplex assay

Frozen supernatants were thawed, and cytokine levels were detected by multiplex assay (LEGENDplex, Biolegend, USA) which is a bead-based immunoassay providing quantification of multiple soluble analytes simultaneously in biological samples using a flow cytometer. The levels of IL-1β, IL-2, IL-4, IL-5, IL-6, IL-8, IL-10, IL-12p70, IL-13, GM-CSF, TNF-α, and IFN-γ were measured according to the manufacturer’s protocol. Acquisition of samples was performed with flow cytometry.

### Statistical analysis and evaluation of data

All flow cytometric measurements were performed on a NovoCyte flow cytometry and analyzed by NovoExpress software (ACEA Biosciences, USA). Statistical analyses were performed with SPSS 21.0 software (SPSS Inc., Chicago, IL, USA). Distribution patterns of subject groups were analyzed by Shapiro–Wilk test. The parametric and non-parametric paired groups were analyzed with Paired-T and Wilcoxon tests, respectively. One-way ANOVA was used to compare multiple groups and post-hoc evaluations were performed by Tukey's test. The values of (*p* < 0.05) were accepted as the statistically significant. Graphs were created using GraphPad Prism 9.0 Software. Data were presented as mean ± standard error mean (SEM).

In order to identify multidimensional differences between the patients at mild, moderate, and severe stages of the disease, correlation network analysis was performed. For this analysis, statistically significant measurements based on the pairwise comparisons followed by Bonferroni adjustment were used. The values were grouped as follows: T cell subsets/intracellular cytokines, NK cells/cytotoxicity, lymphocyte proliferation, cytokines by Multiplex and ELISA measurements. Pearson correlation matrix was created for each category after Z-score standardization. Correlation cut-off was set at ± 0.6 to identify strong non-linear relationships between measurements. Data was visualized by creating a network plot.

## Results

### Diminished T and NK cells in response to C-Vx stimulation

**Figure 1.**
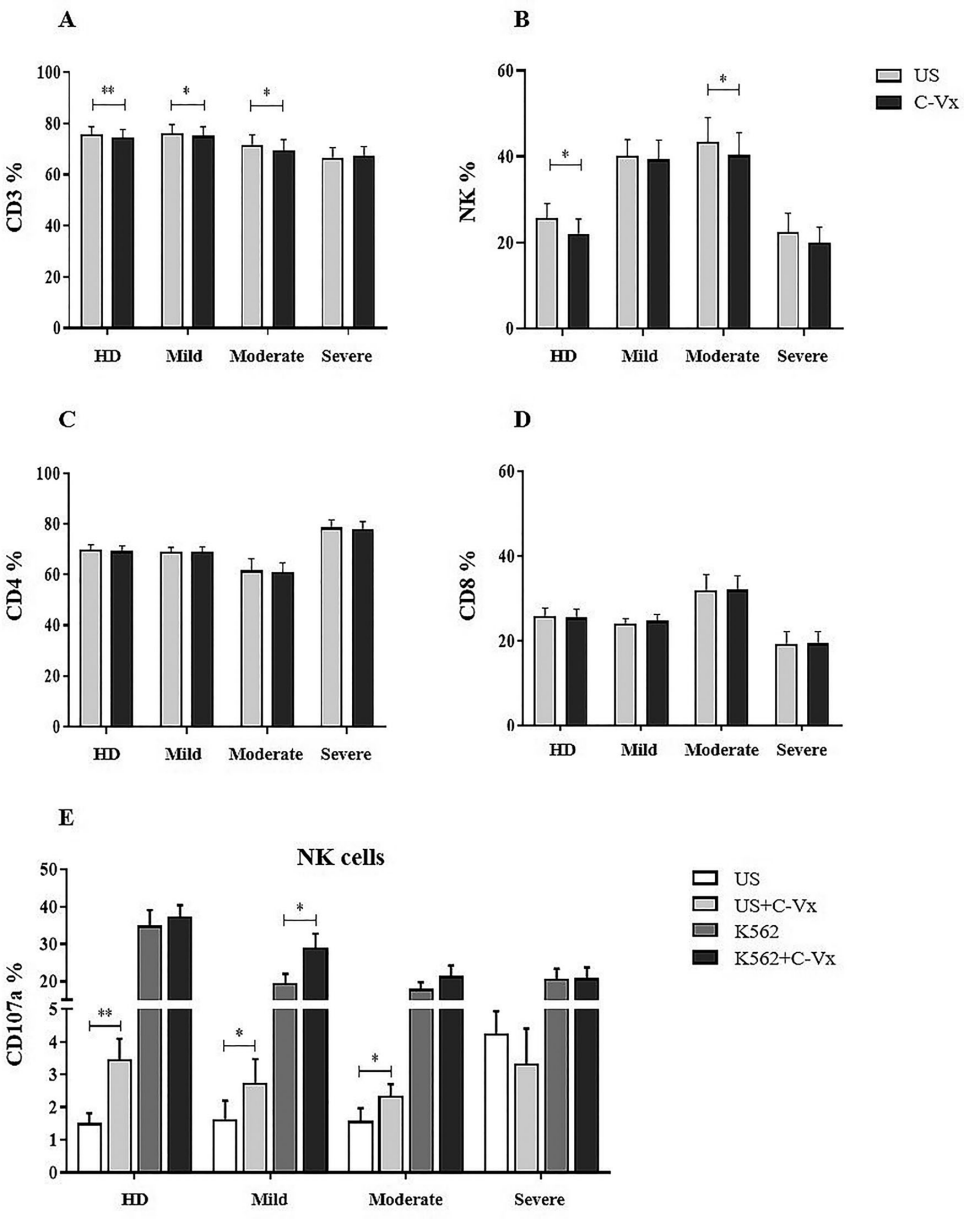
The evaluation of the effect of C-Vx on T and NK cell ratios and NK cell cytotoxicity. (A) CD3^+^ total T, (B) CD3^−^CD16^+^CD56^+^ NK, (C) CD4^+^, (D) CD8^+^ T cell frequencies, and (E) CD107a expression of NK cells in patient groups and healthy donors with and without C-Vx. (*p < 0.05, **p < 0.001, ***p < 0.0001) (US: unstimulated, HD: healthy donors).

Prior to the functional assays, the effects of C-Vx on CD4^+^ and CD8^+^ T cell levels were explored in COVID-19 patients and healthy donors. In all culture assays, 1/250 concentration of C-Vx, determined as the optimal dose as a result of dose experiments, was used (accepted in JCMR). CD3^+^ T cell levels of mild and moderate patients, and healthy donors were significantly decreased upon C-Vx stimulation compared to unstimulated conditions (*p* = 0.022, *p* = 0.023, *p* = 0.008; respectively) (Figure 1(A)). No significant difference was found in CD4^+^ and CD8^+^ T cell ratios of donor groups between conditions with or without C-Vx (Figure 1(C,D)). The reductive effect of C-Vx was also observed on CD3^−^CD16^+^CD56^+^ NK cells. NK cell levels were significantly reduced by C-Vx in moderate patients and healthy donors (*p* = 0.034, *p* = 0.014; respectively) (Figure 1(B)).

### Increased CD107a expression induced by C-Vx

To investigate the effects of C-Vx on the cytotoxic potential of NK and CD8^+^ T cells, the cytotoxicity assay was performed by detection CD107a, perforin and granzyme B levels. No significant difference was found for perforin and granzyme B expression of CD3^−^CD16^+^CD56^+^ NK and CD8^+^ T cells in response to C-Vx, among patients and healthy donors (*p* > 0.05) (data not shown). However, CD107a levels of NK cells were determined to be significantly up-regulated in both patients and healthy donors except in severe patients. In conditions without K562 cells, CD107a expression of NK cells was significantly increased upon C-Vx addition in mild and moderate patients, and healthy donors (*p* = 0.013, *p* = 0.037, *p* = 0.007; respectively) (Figure 1(E)). In the presence of K562, C-Vx addition resulted in a significant elevation only in mild patients (*p* = 0.013). In all donor groups, CD107a expression of CD8^+^ T cells was increased with no statistical significance, in co-culture with K562 cells, regardless of C-Vx stimulation.

### Anti-inflammatory cytokine production in C-Vx-induced T cells

**Figure 2.**
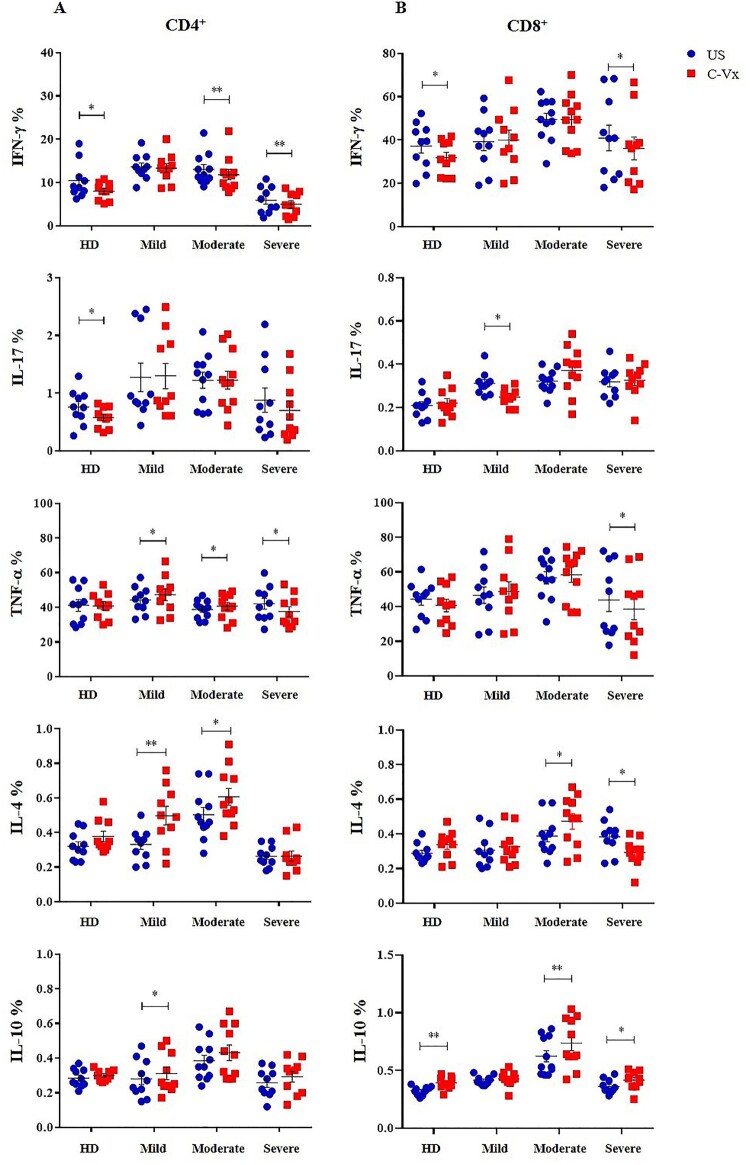
The alterations in intracellular cytokine levels of T cell subsets in response to C-Vx. IFN-γ, IL-17, TNF-α, IL-4, and IL-10 levels of (A) CD4^+^ and (B) CD8^+^ T cells after culture of PBMCs stimulated or not with C-Vx. (*p < 0.05, **p < 0.001, ***p < 0.0001) (US: unstimulated, HD: healthy donors).

In order to evaluate the effects of C-Vx on T cell functions, intracellular levels of IFN-γ, IL-17, TNF-α, IL-4, and IL-10 in CD4^+^ and CD8^+^ T cell subsets were measured upon C-Vx stimulation. PBMCs were pre-cultured with the presence or absence of C-Vx for 72 h, and further stimulated with PMA/Ionomycin prior to the intracellular staining. IFN-γ levels of CD4^+^ T cells were significantly reduced by C-Vx stimulation in moderate and severe patients as well as healthy donors (*p* = 0.008, *p* = 0.003, *p* = 0.026; respectively) (Figure 2(A)). Similarly, IL-17 levels of CD4^+^ T cells were decreased upon C-Vx addition in healthy donors (*p* = 0.01), but not in patients. Nevertheless, the levels of TNF-α were increased following C-Vx stimulation in mild and moderate patients though decreased in severe patients (*p* = 0.029, *p* = 0.036, *p* = 0.033; respectively). In contrast to decreased levels of pro-inflammatory cytokines especially in severe patients, the stimulation with C-Vx increased IL-4 and IL-10 levels, namely Th2-type cytokines, in CD4^+^ T cells. IL-4 content of CD4^+^ T cells was significantly elevated by C-Vx in mild and moderate patients (*p* = 0.001, *p* = 0.013; respectively). The presence of C-Vx led to increased IL-10 levels of CD4^+^ T cells in all patient groups though significance was found only in mild patients (*p* = 0.025).

A similar effect of C-Vx on IFN-γ levels of CD4^+^ T cells was observed on CD8^+^ T cells. IFN-γ^+^CD8^+^ T cell levels of severe patients and healthy donors were significantly reduced by C-Vx addition (*p* = 0.029, *p* = 0.023; respectively) (Figure 2(B)). IL-17 levels of CD8^+^ T cells were similarly decreased after C-Vx stimulation in mild patients (*p* = 0.010). Resembling CD4^+^ T cells, TNF-α content of CD8^+^ T cells in severe patients was found to be decreased by C-Vx (*p* = 0.02). Interestingly, IL-4 levels of CD8^+^ T cells were significantly elevated in moderate patients (*p* = 0.014) whereas reduced in severe patients (*p* = 0.02), in response to C-Vx stimulation. Like CD4^+^ T cells, CD8^+^ T cells also had higher IL-10 levels following C-Vx addition in moderate and severe patients as well as healthy donors (*p* = 0.009, *p* = 0.02, *p* = 0.002; respectively).

### The proliferative effect of C-Vx on lymphocytes

The proliferative responses of lymphocytes to C-Vx stimulation were investigated by the CFSE dilution method. The role of C-Vx on both spontaneous and PHA-induced proliferation of lymphocytes was assayed. The obtained data of CD3^+^, CD4^+^, and CD8^+^ T cells as well as CD3^−^CD16^+^CD56^+^ NK cells were evaluated as % proliferation.

**Figure 3.**
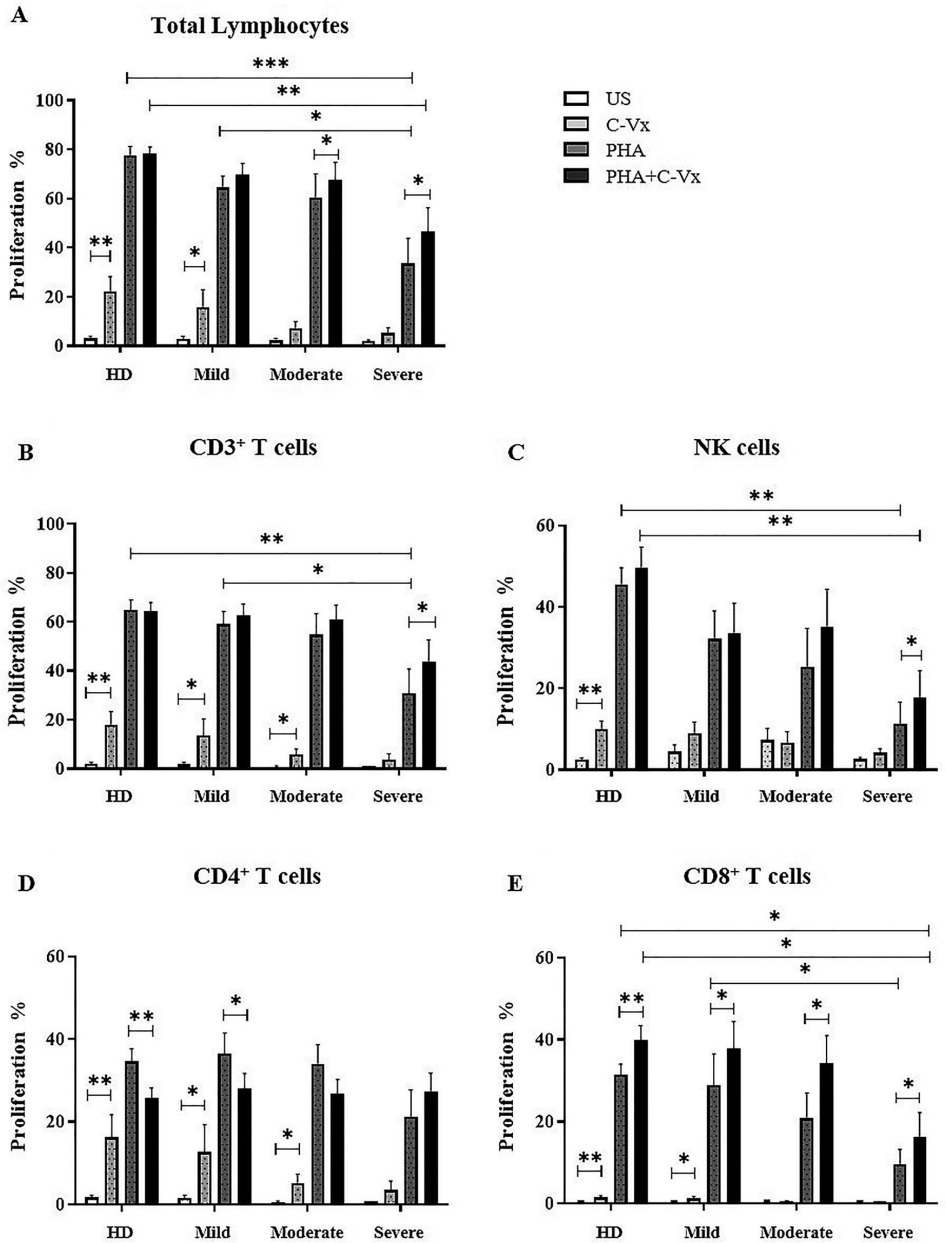
Determination of lymphocyte proliferation in response to C-Vx. The proliferation capacities of (A) total lymphocytes, (B) CD3^+^ T cells, (C) CD3^−^CD16^+^CD56^+^ NK cells, (D) CD4^+^, and (E) CD8^+^ T cell subsets after cell culture with/without PHA stimulation with or without C-Vx. (*p < 0.05, **p < 0.001, ***p < 0.0001) (US: unstimulated, HD: healthy donors).

In total lymphocytes, C-Vx alone induced significant proliferation in mild patients besides healthy donors (*p* = 0.036 and *p* = 0.005; respectively) (Figure 3(A)). This effect declined with increased disease severity. PHA induced strong proliferation, however severe patients had diminished proliferation compared to healthy donors and mild patients (*p* = 0.001 and *p* = 0.029; respectively). The co-existence of C-Vx and PHA triggered total lymphocyte proliferation levels comparable with PHA alone in all patient groups. Notably, diminished PHA-induced proliferation with increased disease severity was regained with the co-existence of C-Vx. In severe and moderate patients, the addition of C-Vx together with PHA triggered stronger proliferation in comparison with PHA alone (*p* = 0.021 and *p* = 0.043; respectively). However, elevated proliferative responses observed in severe patients by the combined stimulation of C-Vx and PHA was lower than in healthy donors (*p* = 0.005).

C-Vx induced the proliferation of CD3^+^ T cells in mild, moderate, and severe patients as well as healthy donors (*p* = 0.012, *p* = 0.028, *p* = 0.028, and *p* = 0.007; respectively) (Figure 3(B)). This effect of C-Vx was observed to decline in concordance with increased disease severity, similar to the case of PHA-induced proliferation. PHA-induced proliferation in severe patients was lower than healthy donors (*p* = 0.007), which was potentiated with the existence of C-Vx (*p* = 0.038). PHA-induced proliferation of severe patients was significantly diminished compared to mild patients (*p* = 0.045).

CD4^+^ T cells responded to C-Vx alone and proliferated consequently in mild and moderate patients, and in healthy donors (*p* = 0.012, *p* = 0.018, and *p* = 0.005; respectively) (Figure 3(D)). No significant difference in PHA-induced proliferation among patient groups and healthy donors was observed, but proliferation in response to a combination of C-Vx and PHA was found to limit PHA-induced proliferation in healthy donors and mild patients (*p* = 0.005 and *p* = 0.012; respectively). In severe patients, C-Vx in addition to PHA was observed to trigger stronger proliferation compared to PHA alone, although without statistical significance.

C-Vx alone induced CD8^+^ T cell proliferation in healthy donors as well as mild patients (*p* = 0.008 and *p* = 0.018; respectively) (Figure 3(E)). Proliferation in response to both PHA and combination of PHA with C-Vx was found to decline with the increased disease severity. PHA-induced proliferation in severe patients was significantly reduced compared to healthy donors (*p* = 0.015). On the other hand, the addition of C-Vx to PHA significantly up-regulated the proliferation levels in mild, moderate, and severe patients as well as in healthy donors (*p* = 0.017, *p* = 0.018, *p* = 0.011, and *p* = 0.013; respectively). However, the proliferative responses against the combination of C-Vx and PHA in severe patients were diminished compared to healthy donors (*p* = 0.019).

CD3^−^CD16^+^CD56^+^ NK cell proliferation in response to C-Vx was significantly increased in response to C-Vx alone only in healthy donors (*p* = 0.005) (Figure 3(C)). The proliferation levels at PHA-induced conditions tended to be decreased with disease severity, while significantly diminished in severe patients, in comparison with healthy donors (*p* = 0.002). A combination of PHA and C-Vx had an up-regulatory effect in comparison with PHA alone, which showed significance only in severe patients (*p* = 0.038). However, in severe patients, PHA and C-Vx together were not able to up-regulate the proliferation to the significantly high levels of healthy donors (*p* = 0.002).

### Bradykinin, IRF3, and IFN-α levels after the stimulation with C-Vx

**Figure 4.**
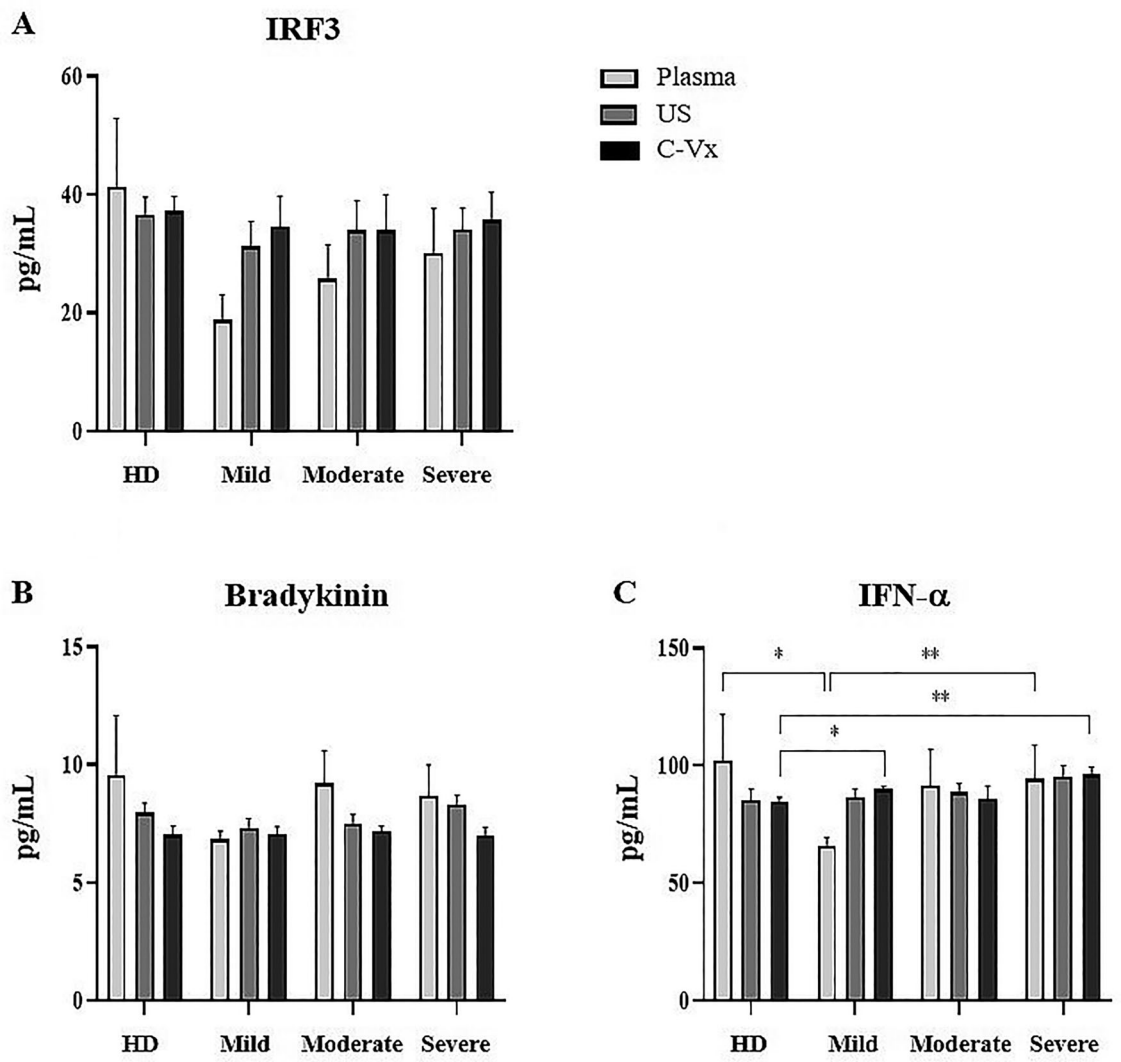
Bradykinin, IRF3, and IFN-α levels in response to C-Vx. The effects of C-Vx addition on (A) IRF3, (B) Bradykinin, and (C) IFN-α levels in plasma and culture supernatants were measured by ELISA method (*p < 0.05, **p < 0.001, ***p < 0.0001) (US: unstimulated, HD: healthy donors).

The levels of bradykinin, IRF3, and IFN-α were measured in plasma samples and supernatants by ELISA. No difference was found in IRF3 and bradykinin levels between donor groups (Figure 4(A,B)). Plasma IFN-α levels were lower in mild patients than severe patients and healthy donors (*p* = 0.007, *p* = 0.035; respectively) (Figure 4(C)). After C-Vx addition, IFN-α levels in supernatants were found to be higher in mild and severe patients compared to healthy donors (*p* = 0.05 and *p* = 0.006; respectively). As a result, the levels of IFN-α, which were low in plasma samples of mild patients, tended to increase after stimulation with C-Vx.

### Elevation of pro-inflammatory cytokines with C-Vx stimulation

**Figure 5.**
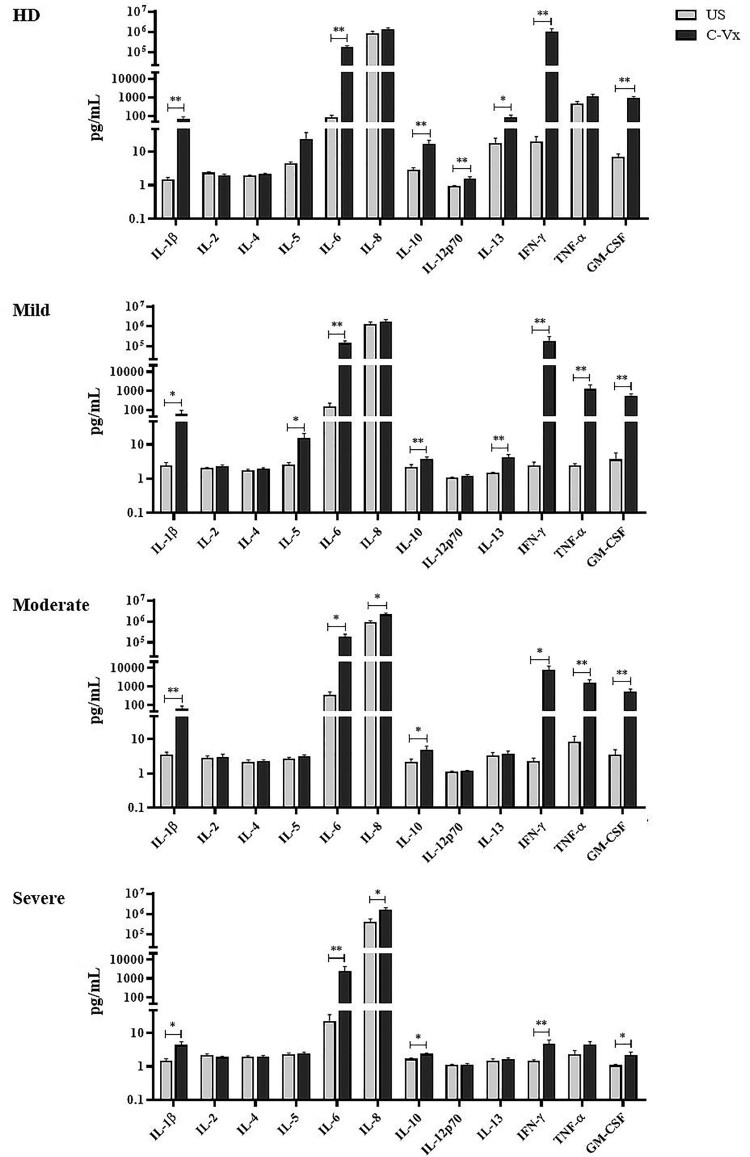
The alterations in cytokine levels after C-Vx addition. IL-1β, IL-2, IL-4, IL-5, IL-6, IL-8, IL-10, IL-12p70, IL-13, IFN-γ, TNF-α, and GM-CSF levels were measured by multiplex assay in supernatants of cultured PBMCs of patients and healthy donors with/without C-Vx. (*p < 0.05, **p < 0.001, ***p < 0.0001) (US: unstimulated, HD: healthy donors).

The levels of IL-1β, IL-2, IL-4, IL-5, IL-6, IL-8, IL-10, IL-12p70, IL-13, IFN-γ, TNF-α, and GM-CSF were measured by multiplex assay in supernatants of PBMCs cultured with/without C-Vx. IL-1β, IL-6, IL-10, IFN-γ, and GM-CSF levels of all donor groups were significantly elevated by C-Vx addition: In mild (*p* = 0.015, *p* = 0.008, *p* = 0.008, *p* = 0.008, *p* = 0.008; respectively), moderate (*p* = 0.008, *p* = 0.012, *p* = 0.017, *p* = 0.017, *p* = 0.008; respectively) and severe patients (*p* = 0.021, *p* = 0.008, *p* = 0.011, *p* = 0.008, *p* = 0.015; respectively) as well as healthy donors (*p* = 0.008, *p* = 0.008, *p* = 0.008, *p* = 0.008, *p* = 0.008; respectively) (Figure 5).

**Figure 6.**
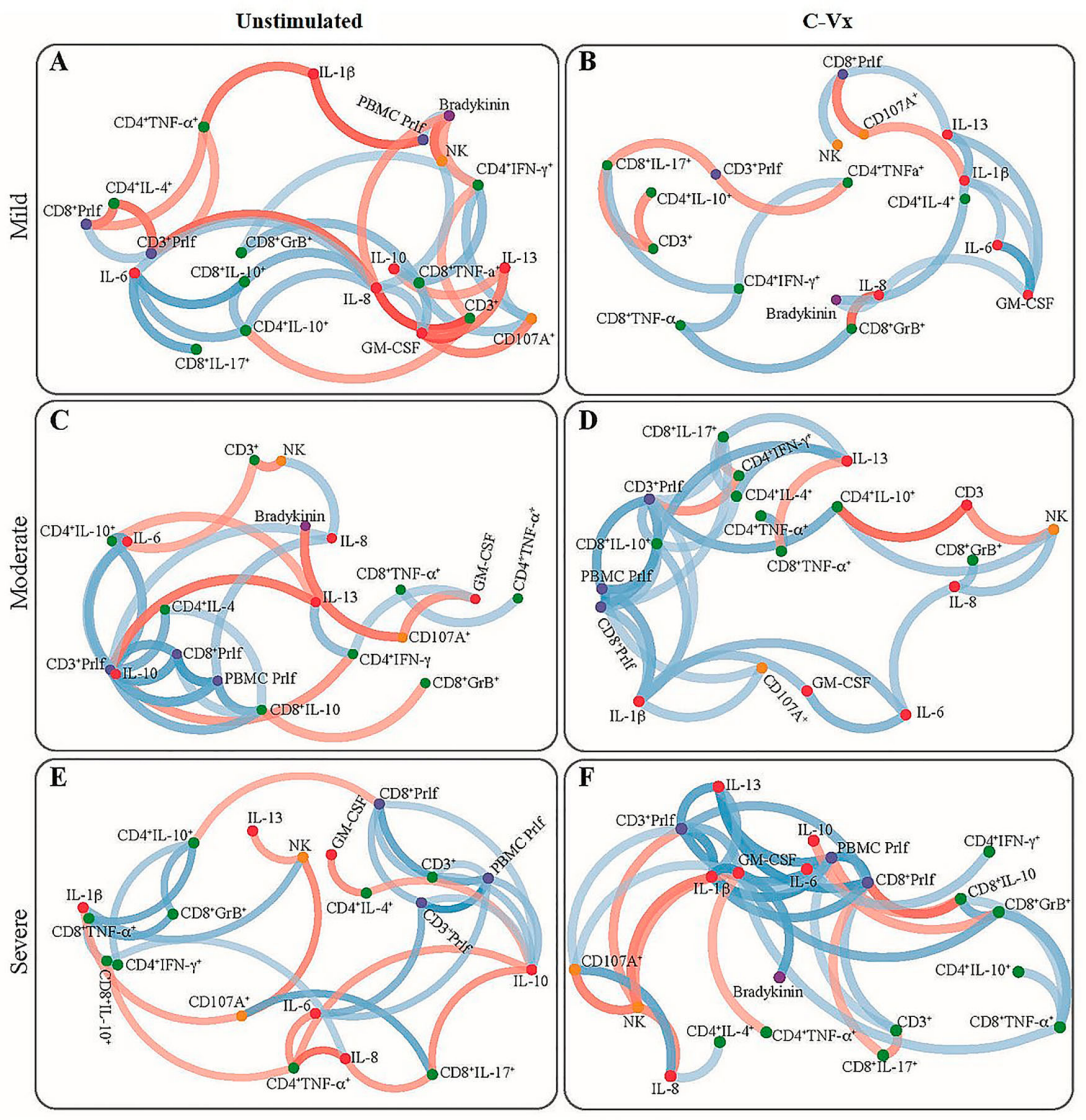
The network analysis of immune parameters in COVID-19 patients with/without C-Vx. Blue and red lines indicate positive and negative correlations. The significance of correlations increases in proportion with increased darkness of line colours. (US: unstimulated).

When correlation analysis was performed for cytokines increased upon C-Vx stimulation, IL-1β levels of mild patients were observed to be positively correlated with IL-6, IL-13, GM-CSF, and bradykinin levels (*p* < 0.05; *r* = 0.65, *r* = 0.74, *r* = 0.62, *r* = 0.71; respectively), whereas a negative correlation was found with CD107a expression of NK cells (*p* < 0.05; *r* = −0.66) (Figure 6). In moderate patients, a positive correlation was detected between the levels of IL-1β, and IL-6, GM-CSF as well as CD107a expression of NK cells (*p* < 0.05; *r* = 0.72, *r* = 0.69, *r* = 0.65; respectively). There was a similar correlation between IL-1β levels of severe patients and GM-CSF, and IL-13 rather than IL-6 (*p* < 0.05; *r* = 0.94, *r* = 0.76; respectively). Moreover, there was a positive correlation between IL-17 levels of CD8^+^ T cells and IFN-γ levels of CD4^+^ T cells in mild and moderate patients (*p* < 0.05, *r* = 0.69 and *p* < 0.05, *r* = 0.64) (Figure 6).

Although IL-13 levels of mild patients and healthy donors were significantly increased at C-Vx-stimulated conditions (*p* = 0.008 and *p* = 0.036; respectively) (Figure 5), there was no difference in moderate and severe patients. However, C-Vx led to a positive correlation between IL-13 and IL-1β levels of severe patients, as presented above, while no correlation was found in unstimulated conditions (Figure 6).

IL-12p70 levels were elevated only in healthy donors (*p* = 0.008) by C-Vx, whereas IL-5 were increased only in mild patients (0.012). TNF-α levels were detected to be significantly higher in supernatants after C-Vx addition in mild and moderate patients (*p* = 0.008, *p* = 0.008; respectively) but not severe patients. In addition, C-Vx stimulation led to an increase of IL-8 levels in moderate and severe patients (*p* = 0.012, *p* = 0.028; respectively). It was noteworthy that IL-1β, IL-6, IFN-γ, GM-CSF, and TNF-α secretion of severe patients were diminished in response to C-Vx in comparison with other donor groups, even though their levels were higher than in unstimulated conditions. Poor response to the stimulation with C-Vx was also observed in IL-10 and IL-13 levels of all patient groups compared to healthy donors. No significant correlation was found among IFN-α, IRF3 levels, and other immune parameters.

## Discussion

The immunomodulatory effects of C-Vx in COVID-19 patients were investigated in a naive patient population who were not previously infected or immunized against COVID-19 by vaccination. Confirming the study showing COVID-19 patients had a low proliferation index of T cells [14], our study demonstrated that PHA-induced proliferation of total lymphocytes, CD3^+^, and CD8^+^ T, and NK cells were diminished in correlation with disease severity. This was partially restored with the presence of C-Vx. As noted in some previous studies [15,16], we observed the proliferation of CD8^+^ T cells was not as strong as in CD4^+^ T cells in COVID-19 patients. Moreover, C-Vx alone did not alter the low proliferative capacity of CD8^+^ T cells in moderate and severe patients. As with CD8^+^ T cells, the effect of C-Vx on cell proliferation was insufficient also in NK cells, which proliferated in response to C-Vx only in healthy donors.

Overall, C-Vx seems to be an effective agent inducing the proliferation particularly of CD3^+^ and CD4^+^ T cells in healthy donors and patients with mild to moderate COVID-19. The decreased proliferative responses in harmony with disease severity might indicate a disrupted immune function in severe COVID-19 forms which could be re-gained by C-Vx.

NK cells are innate immune responders with their critical contributions to virus clearance and immunomodulation. Together with NK cells, CD8^+^ T cells also play an important role in immune defense against viral infections [17,18]. The cytotoxicity of CD8^+^ T and NK cells were revealed to have an exhausted phenotype in patients with COVID-19 [19,20]. CD107a (LAMP-1) is considered as a cell surface marker of activated CD8^+^ T and NK cell degranulation [21]. In this study, the cytotoxic capacity of CD8^+^ T and NK cells in COVID-19 patients and healthy donors were investigated by detecting perforin, granzyme B, and CD107a expression of these cells. Although there was no significant difference before and after C-Vx stimulation in the intracellular perforin and granzyme B levels of CD8^+^ T and NK cells, increased expression levels of CD107a were evaluated in NK cells of patients and healthy donors, except for severe cases. Nevertheless, in patients infected with SARS-CoV-2, CD107a expression was observed to be decreased in comparison with healthy donors, and C-Vx did not seem to alter the weak response to K-562 cells much.

IFN-α is a natural anti-viral cytokine. Decreased production or dysfunction of IFN-α has been presumed to be associated with an increased propensity to COVID-19 infection [22]. Therefore, it was thought that a possible IFN-α-enhancing effect of C-Vx stimulation could pave the way for the use of this agent as a prophylactic treatment method in the prevention of COVID-19 infection. However, in this study, the levels of IFN-α in plasma and culture supernatants were found to be similar among patient groups and healthy donors. Nevertheless, higher IFN-α levels were observed in mild and severe patients than healthy donors, after C-Vx stimulation.

As a pro-inflammatory cytokine, IFN-γ has immunoregulatory roles in improving anti-viral immune responses and limiting excessive reactions [23]. Insufficient IFN-γ production was shown to elevate the replication of viruses and cause decreased survival in viral infections [24,25]. IFN-γ production of antigen-activated T lymphocytes plays critical roles in anti-viral responses and provides a long-term immune defense in viral infections by coordinating the other immune components [26,27]. Moreover, IFN-γ provides the differentiation and maturation of T and B cells involved in the regulation of anti-viral responses [28–30]. Gadotti et al. identified IFN-γ as a risk factor for mortality in moderate and severe COVID-19 patients [31]. In our study, IFN-γ levels of CD4^+^ and CD8^+^ T cells were found to be decreased in all donor groups except mild patients after C-Vx stimulation. However, with the presence of C-Vx, superior levels of IFN-γ were detected in supernatants of cultured PBMCs. This finding indicates C-Vx might have a beneficial effect for anti-viral defenses in COVID-19 by inducing IFN-γ production. Notably, although IFN-γ levels of all patient groups were diminished compared to healthy donors, the IFN-γ-inducing effect of C-Vx was weak only in severe patients.

Similar findings were observed for IL-1β, IL-6, and GM-CSF levels, which were increased by C-Vx stimulation even though being less in severe patients. IL-1β and IL-6, pro-inflammatory cytokines having critical roles in infection and inflammation, have been found to be associated with severe lung injury during influenza A (H1N1) virus infection [32]. In this study, the levels of IL-1β and IL-6 were elevated in mild and moderate patients compared to healthy donors, however, they were observed to be suppressed in severe patients even after C-Vx stimulation.

GM-CSF is a stimulant for multipotent progenitor cells depending on its concentration and is also known to have pro-inflammatory roles during inflammation [33]. In clinical studies, GM-CSF treatment has been demonstrated to be beneficial for patients with sepsis who are especially with severe immunosuppression [34]. In this study, C-Vx stimulation increased GM-CSF levels of mild and moderate patients, and healthy donors. However, the levels of GM-CSF as a response to C-Vx were insufficient in severe patients. IL-10 is an anti-inflammatory cytokine with immunoregulatory functions in viral infections [35]. In this study, the stimulant effect of C-Vx for inducing IL-10 production was observed to be decreased in mild to severe patient groups compared to healthy donors.

Overall, C-Vx stimulation, which nearly did not alter IL-2, IL-4, IL-5 or IL-13 levels, seems to stimulate cytokine production toward Th1 instead of Th2 response. The contrasting profile of intracellular cytokine levels of CD4^+^ and CD8^+^ T cell subsets suggests T cells might not be the main source of the elevation in pro-inflammatory cytokines. On the other hand, these results demonstrated the production of pro-inflammatory or immunoregulatory cytokines was inadequate in the severe phase of COVID-19 even with C-Vx. Thus, the C-Vx application might be more successful in the mild and moderate stages of the disease. In a recent study of our group including mild, moderate, and severe patients, increased anti-inflammatory but decreased inflammatory responses were correlated with disease severity [36]. Supporting these results, the correlation analysis in this study demonstrated more positive or negative correlations in the Th2 direction without C-Vx, which increase from mild to severe phase. However, the correlation clustering was mostly in the direction of Th1 in C-Vx-stimulated conditions. In addition, it should be noted that correlation network plots show a correlation between positive or negative increases or decreases rather than a causality.

Consequently, there is a necessity to perform well-designed, randomized clinical trials in a settings environment of SARS-CoV-2 infection, which will pave the way for evidence-based therapeutics for the worldwide pandemic. Investigation of C-Vx as a patented immunomodulatory agent focused on strengthening the immune system of COVID-19 patients, revealed that C-Vx could be a treatment option for patients especially at mild-to-moderate stages. Further functional studies with C-Vx would clarify the contribution of this novel substance to anti-viral responses against SARS-CoV-2.
